# Retraction: Community’s perceived high risk of coronavirus infections during early phase of epidemics are significantly influenced by socio-demographic background, in Gondar City, Northwest Ethiopia: A cross-sectional -study

**DOI:** 10.1371/journal.pone.0347175

**Published:** 2026-04-16

**Authors:** The PLOS One Editors

After this article [1] was published, concerns were raised about the data used in this study.

Specifically:

- The article [1] reports the same study and data set as [2,3, retracted in 4] and [5, retracted in 6], which were published after [1].
- The description of the data collection tools described in “Data collection tools and variable measurement” does not align with the copy of the survey questionnaire provided in the S1 Appendix. It is therefore unclear how all variables were measured. When asked to clarify how questionnaire variables were measured, the responses did not resolve the question.
- The article does not provide justification for dichotomization of knowledge and attitude variables. The authors indicated that this decision was made to enhance interpretability and public-health relevance of the findings. However, concerns remain about the validity and generalizability of this approach in the absence of any formal validation methods.
- The data are missing headers, and the authors did not provide a version which could be used to replicate the analysis.

In light of the above concerns, the *PLOS One* Editors retract this article.

GGK did not agree with the retraction. MA, THM, SDW, JA, TA, ZNA, MWM, AGM, DMG, GMK, MKY, SYT, KAG, HSM, AWA, CAW, GMB, NTA, CDA, TA, ATT, BKR, EBT, AAT, ZA, HD, and KTG either could not be reached or did not respond directly.
